# Changes in Length and Complexity of Clinical Practice Guidelines in Oncology, 1996-2019

**DOI:** 10.1001/jamanetworkopen.2020.0841

**Published:** 2020-03-13

**Authors:** Benjamin H. Kann, Skyler B. Johnson, Hugo J. W. L. Aerts, Raymond H. Mak, Paul L. Nguyen

**Affiliations:** 1 Department of Radiation Oncology, Dana-Farber Cancer Institute, Brigham and Women’s Hospital, Harvard Medical School, Boston, Massachusetts; 2 Artificial Intelligence in Medicine Program, Brigham and Women’s Hospital, Boston, Massachusetts; 3 Huntsman Cancer Institute, The University of Utah, Salt Lake City

## Abstract

This cross-sectional study calculates the increase in clinical cancer knowledge represented in the National Comprehensive Cancer Network (NCCN) Clinical Practice Guidelines from 1996 to 2019.

## Introduction

The last century has seen substantial increases in the generation of knowledge pertaining to clinical cancer care.^1^ In 1995, the National Comprehensive Cancer Network (NCCN), a not-for-profit alliance of National Cancer Institute–designated cancer centers, was created with the goal of developing standards of care for cancer treatment. Since its inception, the NCCN has published regularly updated Clinical Practice Guidelines in Oncology (NCCN Guidelines) that cover recommended management for 97% of patients with cancer. The guidelines are used by as many as 95% of oncologists in the US in clinical practice and are relied on by public and private insurers to determine oncology coverage policies.^2^ Since the establishment of the NCCN Guidelines, cancer management options have continued to increase in quantity and complexity because of advances in personalized medicine, novel therapeutics, and technology. The extent to which cancer data and knowledge are growing has implications for policy making, medical education, and the health care system. Thus, we sought to quantify the increase in clinical cancer knowledge represented in the NCCN Guidelines since the turn of the 21st century.

## Methods

We analyzed current and archived NCCN Guidelines from 1996 to 2019 using the NCCN website, the Internet Archive,^3^ and the Harvard Medical School library. The study follows the Strengthening the Reporting of Observational Studies in Epidemiology (STROBE) reporting guideline for cross-sectional studies and was conducted with permission from the NCCN. Because the study does not involve human participants, it was exempt from institutional review board review per the Common Rule. We accessed NCCN Guidelines in original electronic portable document format and performed the data analysis during September 2019. The most common cancers (ie, breast, prostate, non–small cell lung, and colon) were selected for analysis.

Document page count was evaluated as a surrogate for guideline knowledge volume. To characterize document complexity, we evaluated total references cited in the discussion section of each guideline document as well as the number of decision paths present in the flowchart sections of guideline documents from 1996 and 2019. A *decision path* was defined as a unique guideline path resulting from forks in the guideline flowchart for a particular subtype of malignant neoplasm. We modeled PC and RC trends via linear, polynomial, and exponential regressions for best fit. Analysis was conducted by disease site and averaged. For each year, standard deviation among disease sites was calculated. Statistical analyses were conducted using Stata version 13 (StataCorp). Given that statistics were mainly descriptive, no threshold for statistical significance was prespecified.

## Results

**Figure 1.**
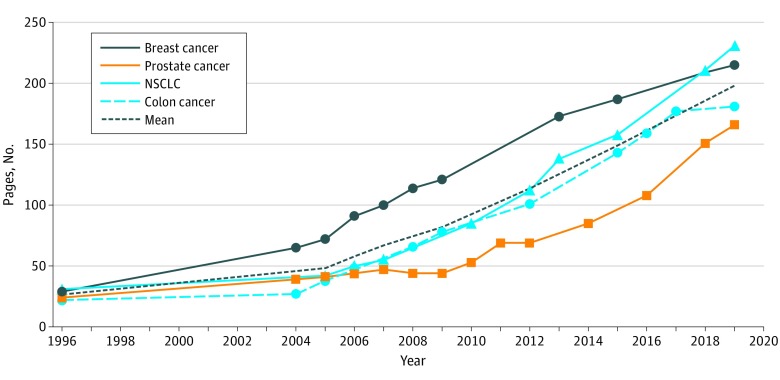
Page Volume of National Comprehensive Cancer Network Clinical Practice Guidelines by Disease Site, 1996-2019 NSCLC indicates non–small cell lung cancer.

**Figure 2.**
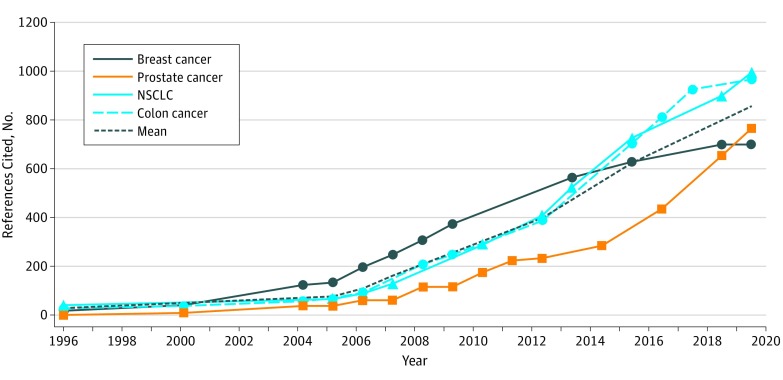
References Cited in National Comprehensive Cancer Network Clinical Practice Guidelines by Disease Site, 1996-2019 NSCLC indicates non–small cell lung cancer.

Between 1996 and 2019, the mean (SD) page count of NCCN Guidelines increased from 26 (4.2) to 198 (30.0) pages, a 762% absolute increase overall and a mean increase of 7.5 pages annually (Figure 1). Mean (SD) references cited increased from 28 (16.8) to 856 (146.3), a 3057% increase overall and a mean increase of 36 references annually (Figure 2). Similar increases were seen across all cancer types studied. The mean (SD) number of decision paths increased from 30 (8.5) to 111 (49.5), a 370% absolute increase. Trends in page count and references cited were best fit by exponential regression (*R*^2^ = 0.99 for both). Using the best-fit models, projections for mean page count and references cited in 2025 would be 355 pages and 1954 references per disease site guideline.

## Discussion

Clinical practice guidelines in oncology have increased dramatically in quantity and complexity over the past 2 decades and follow an exponential growth pattern. Recent and projected guideline growth, which is associated with both the accumulation of new data and expert opinion, has implications for oncology trainees, policy makers, payers, and patients. Study limitations include unavailable archived guidelines from certain years. Furthermore, while the metrics used represent interpretable surrogates, they may not be directly proportional to document complexity. If increases continue exponentially, the amount of data assimilation required to deliver optimal care may be unsustainable for individual practitioners.^4,5^ Strategies to organize oncologic knowledge, such as the creation of Drugs and Biologics and Imaging Compendia,^6^ have been implemented. Further approaches, including guideline stratification by evidence level and the use of artificial intelligence for decision support, should be investigated as ways to synthesize data and improve cancer decision-making.
